# Neutrophil interactions with the sexually transmitted parasite *Trichomonas vaginalis:* implications for immunity and pathogenesis

**DOI:** 10.1098/rsob.200192

**Published:** 2020-09-02

**Authors:** Suhani B. Bhakta, Jose A. Moran, Frances Mercer

**Affiliations:** Department of Biological Sciences, California State Polytechnic University, Pomona, CA, USA

**Keywords:** neutrophil, *Trichomonas vaginalis*, trogocytosis, *Mycoplasma hominis*, inflammation, sexually transmitted infection

## Abstract

Trichomoniasis is the third most common sexually transmitted infection in humans and is caused by the protozoan parasite, *Trichomonas vaginalis* (*Tv*). Pathogenic outcomes are more common in women and generally include mild vaginitis or cervicitis. However, more serious effects associated with trichomoniasis include adverse reproductive outcomes. Like other infectious agents, pathogenesis from *Tv* infection is predicted to be the result of both parasite and host factors. At the site of infection, neutrophils are the most abundant immune cells present and probably play key roles in both parasite clearance and inflammatory pathology. Here, we discuss the evidence that neutrophils home to the site of *Tv* infection, kill the parasite, and that in some circumstances, parasites possibly evade neutrophil-directed killing. *In vitro*, the parasite is killed by neutrophils using a novel antimicrobial mechanism called trogocytosis, which probably involves both innate and adaptive immunity. While mechanisms of evasion are mostly conjecture at present, the persistence of *Tv* infections in patients argues strongly for their existence. Additionally, many strains of *Tv* harbour microbial symbionts *Mycoplasma hominis* or *Trichomonasvirus*, which are both predicted to impact neutrophil responses against the parasite. Novel research tools, especially animal models, will help to reveal the true outcomes of many factors involved in neutrophil-*Tv* interactions during trichomoniasis.

## 1. Introduction

*Trichomonas vaginalis* (*Tv*) is a human-specific extracellular, flagellated protozoan parasite responsible for the third most common sexually transmitted infection (STI) in the United States (US) and worldwide, called trichomoniasis [1–3]. Worldwide, trichomoniasis case numbers approach 400 million, making it the most common non-viral sexually transmitted infection [4]. Despite the high prevalence of *Tv* infection, trichomoniasis is classified as a neglected infectious disease in the US owing to its high prevalence and the relative lack of research regarding the infection [3]. It is commonly treated with 5-nitroimidazole drugs such as metronidazole or tinidazole. Unfortunately, antibiotic-resistant *Tv* strains are on the rise, making treatment of some infections difficult [1]. However, no other treatment options are currently approved to treat or prevent trichomoniasis [1].

*Tv* attacks the host by attaching to, and often subsequently killing cells in the urogenital tract, such as cervicovaginal and prostate epithelial cells [5,6]. The process of *Tv* attachment to host cells is called cytoadherence [6,7]. During cytoadherence, the parasite alters its usually pear-shaped morphology to adopt an amoeboid form, increasing host cell surface area coverage [5,8], which is postulated to aid in the parasite's retention within the host [5,8]. It is also posited that the parasite obtains nutrients through the destruction of host epithelial cells [5]. The degradation of cervicovaginal epithelial cells is thought to be the source of vaginitis and colpitis macularis (commonly referred to as ‘strawberry cervix') [9]. Other adverse effects may include pelvic inflammatory disease and infertility [10–12]. Infection during pregnancy is associated with pre-term delivery, causing low birth weight infants, putatively owing to early rupture of the uterine membrane [10,11,13,14]. While *Tv* infection is overwhelmingly asymptomatic in men, some patients may experience penile discharge, discomfort during urination or irritation in the urethra [15]. Trichomoniasis also contributes to the spread of human immunodeficiency virus (HIV), as incidences of HIV have been found to be higher in *Tv*+ populations [11,16,17]. Epithelial cell damage caused by *Tv* may also allow for increased malignancy of cervical neoplasms, as later grades of cervical cancers were found to be increased in human papillomavirus+ patients that are co-infected with *Tv* [18–20]. One major factor in the broad spectrum of disease severity associated with trichomoniasis is likely to be the strain of parasite, because clinical isolates vary broadly in their ability to kill cervicovaginal and prostate epithelial cells *in vitro* [6]. However, host factors such as the individualized microbiome [21,22], and immune response most likely also play a role [7]. In particular, many of the aforementioned symptoms are linked to inflammation [10].

Innate immune cells called neutrophils are considered to be the major player in *Tv*-associated inflammation, as they are the most inflammatory cells in the immune system and are abundantly recruited to the vagina during *Tv* infection [23]. Neutrophils are also the most abundant immune cell type in the blood [24] and are the first cells recruited to the site of most infections, as they extravasate from the blood in large numbers, responding to local inflammatory cues [24–26]. Once in the infected tissue, their effector functions serve to quickly and efficiently kill pathogens to reduce their dissemination [24,26]. A white-frothy discharge rich in neutrophils has long been a clinical hallmark of trichomoniasis [9]. In trichomoniasis patients, neutrophils are abundant in wet mount smears from vaginal discharges and penile urethral samples [23,27,28]. Furthermore, the quest to establish a mouse model to study trichomoniasis has been stymied by the large influx of neutrophils to the vagina following inoculation with *Tv*, arguing for both the recruitment of neutrophils to the infection and also their ability to kill the parasite [29]. However, while this evidence supports that neutrophils play important roles in clearing *Tv*, neutrophils are also well-known to cause myriad inflammatory pathologies [30] and could therefore also drive many symptoms and sequelae of trichomoniasis. Therefore, whether the cumulative impact of neutrophil activity during trichomoniasis is beneficial or detrimental to the host is unknown, and most likely depends on many variables that occur during natural infection. Undoubtedly, however, a better understanding of the actions of neutrophils during trichomoniasis is important for understanding the pathogenesis of and immunity to *Tv*.

## 2. Neutrophil homing to *Tv* infection

**Figure 1.**
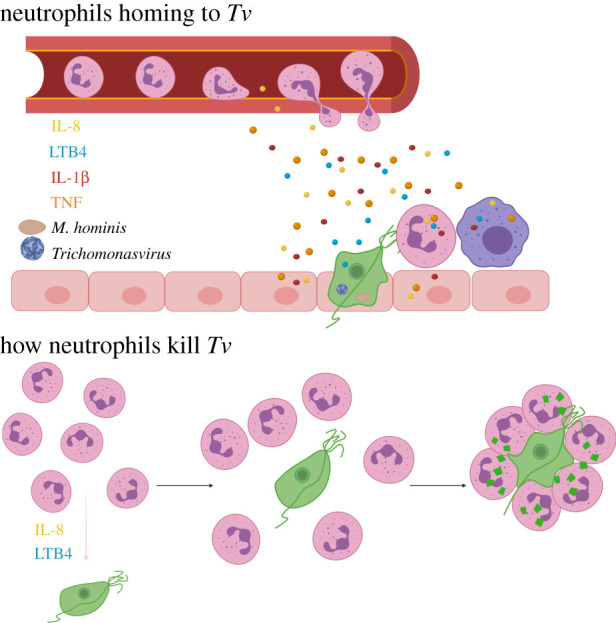
Neutrophils flow rapidly through blood, but activation of the endothelium by inflammatory mediators near an infection site can begin the process of neutrophil extravasation through diapedesis. Inflammatory mediators are secreted in response to *Tv* (green) from epithelial cells (pink), resident macrophages (purple) and neutrophils that arrive first (pink cells with multi-lobed nuclei). The greatest amount of inflammatory cytokines is triggered by strains of *Tv* that harbour symbionts *Mycoplasma hominis* or *Trichomonasvirus*. Once out of the blood, neutrophils follow IL-8 and LTB4 gradients to home to parasites. Upon encountering *Tv*, neutrophils swarm and trogocytose the parasite.

Neutrophils probably home to the site of *Tv* infection following cues from both the parasite itself and host-produced factors (figure 1). The first cells to encounter the parasite during the initial stage of infection are the urogenital epithelial cells that the parasite attaches to (described above), as well as tissue-resident macrophages and dendritic cells [31]. Responding to cues from cells at the infection site, neutrophils rapidly leave the blood to infiltrate infected tissues, in a process known as extravasation [25]. Therefore, it is useful to consider neutrophil homing to *Tv* in two phases: (i) extravasation from the blood into the infected tissue, and (ii) homing to individual parasites once in the infected tissue. Importantly, both of these processes are predicted to be influenced by the presence of microbial symbionts within *Tv*.

### 2.1. Extravasation and homing to individual parasites

The process of extravasation is mediated by adhesive molecules on both the neutrophil and vascular endothelial cells. First, selectins are upregulated on vascular endothelial cells in response to local inflammatory cytokines such as interleukin (IL)-1*β* and tumour necrosis factor (TNF) [32]. Neutrophils flow rapidly through the blood, however, when they pass through capillaries where endothelial cells have upregulated selectins, the neutrophils decelerate and roll along capillary walls, owing to the neutrophil's constitutively expressed low-affinity selectin ligands [25]. Following this rolling, neutrophil activation occurs, both by signalling through the selectin ligand, and by inflammatory cytokines in the area such as IL-8 [25]. Neutrophil activation results in the expression of the active-confirmation integrin that binds intercellular adhesion molecules (ICAMs) on the surface of the endothelial cells with high affinity, causing a halt [25]. The neutrophil then passes through the endothelium at the cell–cell boundaries and also penetrates the basement membrane, entering the tissue: a process known as diapedesis [25]. Leukotriene chemical mediators have also been shown to activate endothelium and increase vascular permeability to promote extravasation and diapedesis [33]. Therefore, inflammatory cytokines, such as IL-1*β*, TNF and IL-8, and leukotrienes secreted at the site of *Tv* infection are hypothesized to be important in neutrophil extravasation from the blood into the tissue.

Once in the tissues, neutrophils continue to follow chemotactic cues to home directly to pathogens. Neutrophils also exhibit ‘swarming motility,' attacking pathogens in aggregate [25]. In the case of *Tv*, visualization of vaginal smears shows neutrophils following the parasite in swarms [34], an observation that we also made with axenically grown trichomonads co-cultured with neutrophils isolated from peripheral blood [35].

Early work showed that *Tv* produces a chemotactic factor that attracts neutrophils [36]. This factor was found to be leukotriene-B4 (LTB4), an eicosanoid lipid mediator that plays roles in promoting extravasation [33,36]. In addition to parasite-produced LTB4, neutrophils themselves make LTB4, perpetuating a positive feedback loop once the first neutrophil has encountered a trichomonad, signalling for more recruitment to the infected area [37]. LTB4 has also been detected at the site of *Tv* infection in vaginal discharges, supporting its involvement during natural infection [38,39]. Analyses also showed that higher levels of LTB4 at the infection site were correlated with symptomaticity [39], indicating the role of LTB4 in modulating inflammation. Patients with higher LTB4 levels also had more neutrophils at the infection site, supporting a role for neutrophils in pathogenesis [39]. As neutrophils greatly outnumber trichomonads in vaginal discharges (greater than 100 : 1) [23], the major source of LTB4 during infection is likely to be neutrophils. It is not clear, however, whether LTB4 production and secretion by *Tv* itself confer any advantage to the parasite in the host, but its conservation, despite attracting neutrophils, points to a potentially redundant function for this eicosanoid in an essential function in *Tv* biology.

*Tv* has also long been known to induce IL-8 secretion from host cells [40–42] and has been demonstrated to stimulate IL-8 from a variety of cell types that are present at the site of initial infection such as epithelial cells and macrophages [41,43–46]. IL-1*β* and TNF have also been shown to be induced from host cells by the parasite [43–46], supporting a model that local cytokine responses from epithelial cells and resident macrophages during initial *Tv* infection can activate both the local endothelium and neutrophils within the blood to promote extravasation of neutrophils into the infected tissue. Once in the tissue, IL-8 is thought to be the most important chemokine for neutrophil homing to the parasite, as IL-8 is the most potent neutrophil-recruitment chemokine [47] and is consistently found to be abundantly secreted from host cells following *Tv* challenge [43–45]. Interestingly, patients infected with *Tv* that displayed symptoms had a higher neutrophil presence and IL-8 concentrations, compared to those who were asymptomatic [41], again supporting the notion that neutrophils may play a role in pathogenesis.

Furthermore, the protein C5a, which is generated when the complement system (described below) is activated, also aids in diapedesis and serves as a chemoattractant for neutrophils [25,48]. As *Tv* is known to activate complement [49], C5a may also contribute to neutrophil homing to *Tv* during infection.

### 2.2. The role of microbial symbionts in *Tv* immune activation

Many *Tv* strains harbour microbial symbionts that are likely to strongly promote neutrophil infiltration and attraction, by triggering increased inflammatory cytokine responses. IL-8, IL-1*β* and TNF were all markedly increased in instances where the parasite was harbouring either of its two microbial endosymbionts, *Mycoplasma hominis* or *Trichomonasvirus*. Human *M. hominis* is an obligate parasitic bacterium with a minimal genome and limited biosynthetic abilities [50–52]. While it can be detected in the vaginal microflora of healthy women [53], it is more commonly found in women with bacterial vaginosis (BV) [53,54] and is associated with pre-term birth [55,56]. While *M. hominis* can invade host epithelial cells [57,58], intriguingly, the bacteria can also reside within *Tv* as a symbiont [50]. *Trichomonasvirus* is a double-stranded RNA (dsRNA) virus in the totiviridae family [59,60]. Both symbionts are highly prevalent in clinical isolates of *Tv*, although their prevalence among strains varies by geographical region. The prevalence and other aspects of the symbionts' biology and contribution to pathogenesis are nicely reviewed elsewhere [51,61]. For the purposes of this review, however, it is important to note that the presence of the symbionts greatly impacts which pattern-recognition receptors (PRRs) are stimulated on host cells, since *Tv*, *M. hominis*, and *Trichomonasvirus* all have distinct pathogen-associated molecular patterns (PAMPs) [62]. PRR engagement is a strong determinant of the quantity and quality of cytokine and chemokine secretion [62].

The major PAMP on *Tv* is currently thought to be the dominant surface lipoglycan (LG) (also referred to as the lipophosphoglycan (LPG)), an abundant *Tv* glycoconjugate that coats the surface of the parasite [63,64], and binds to host cell galectins 1 and 3, resulting in cytokine production [65]. *Trichomonasvirus* may conceivably trigger either of the known PRRs that recognize dsRNA, Toll-like receptor 3 (TLR3), and/or the RIG-I-like receptors RIG-I and MDA5 [66]. Data currently supports that *Trichomonasvirus* engages TLR3 when *Tv* strains containing *Trichomonasvirus* are used to stimulate host cells [45]. *Mycoplasma hominis* is thought to activate host cells through TLR2, as is common for mycoplasmas [67,68]. Therefore, the presence of the symbionts expands the PRR- triggering capacity of the parasite. Lysis of the parasite, which occurs from antibiotic treatment, was shown to promote increased cytokine production from host cells, presumably owing to increased release of intracellular symbionts into the extracellular space, where they can more easily access host cell PRRs [45]. Therefore, the symbionts may have an even greater effect on host cell cytokine secretion *in vivo* when the parasite is under assault by neutrophils, potentially promoting the increased release of the symbionts from lysed parasites.

In studies where we compared strains harbouring or cleared of *M. hominis*, IL-1*β* was completely absent and IL-8 was severely (greater than fivefold) reduced, when monocytes were stimulated by *M. hominis*-free strains [43], showing that *M. hominis* is probably responsible for a majority of inflammatory cytokine secretion induced by the parasite. In similar experiments using a myeloid cell line, Fiori *et al*. [44] also observed substantial increases in IL-1*β* and TNF when *M. hominis* was added to *Tv*, and in fact, TNF was completely absent when strains without the addition of *M. hominis* were used. Fichorova *et al*. [45] compared cytokine responses from epithelial cells in *Trichomonasvirus+* versus *Trichomonasvirus-* strains and similarly observed increases in IL-1*β* and IL-8 induced from strains that harbour the virus compared to those that do not. Collectively, these data support that a major factor in activating host cells to produce neutrophil-recruitment factors may actually be ligands from *Tv*'s symbionts rather than the parasite itself. Therefore, whether the infecting strain of the parasite harbours either or both of these symbionts probably has major impacts on pathogenesis.

## 3. Mechanism of neutrophil killing of *Tv*

Once neutrophils home to *Tv*, it is conceivable that they could either succeed or fail at killing the parasite, and clearing infection. In either scenario, neutrophil activity could result in collateral damage to host tissues and therefore contribute to pathogenesis. However, if neutrophils succeed at killing *Tv*, this may outweigh or limit the duration of any collateral damage. In this section, we will discuss supporting evidence that neutrophils kill *Tv*, and we discuss potential evasion mechanisms further on in this review. However, we acknowledge that during a natural infection, multiple variables exist that may contribute to the outcome, including the strain, whether symbionts are present, host immune variability, and also the cervicovaginal microbiome (CVM).

### 3.1. How neutrophils kill

Neutrophils have three main killing mechanisms used against pathogens: phagocytosis, extracellular degranulation and NETosis [69]. Phagocytosis is the engulfment of whole pathogens and is followed by subsequent digestion of the pathogen when the phagosome fuses either with a lysosome, or with neutrophil toxic granules. Neutrophil toxic granules are organelles that contain antimicrobial contents such as pore-forming toxins, proteases and reactive chemical species. Degranulation is the exocytosis of these toxic granules from neutrophils, where they may intoxicate pathogens in the extracellular space. NETosis, short for Neutrophil Extracellular Traps, is the ejection of DNA, histones and toxic granules from neutrophils into the extracellular space, resulting in pathogen ensnarement in ‘NETs' of unravelled DNA. The immobilized pathogens are then subject to toxic granules and/or nearby phagocytic cells [69]. Importantly, while phagocytosis and extracellular degranulation ensue rapidly after pathogen encounter, NETs generally take 2–4 h to deploy [69]. It is not fully clear whether neutrophils receive a signal to determine which mechanism to employ following sensing of the pathogen, or whether they attempt phagocytosis and degranulation first, and NETosis ensues later if the earlier mechanisms are not productive. However, intriguing work using *Candida albicans* and *Mycobacterium bovis* showed that neutrophils phagocytosed small yeast and individual bacteria while they NETosed hyphae and large bacterial aggregates and showed that signalling downstream of successful phagocytosis downregulated the NETosis machinery, pointing to a size-sensing mechanism that neutrophils may broadly use to regulate the employment of discrete killing mechanisms [70]. It is also possible that the three mechanisms are not mutually exclusive, but that neutrophils eventually use a combination of the three during a particular infection. This is a logical hypothesis, as many pathogens may be morphologically heterogenous *in vivo*, with some pathogens existing as individuals, and others in biofilm form. Furthermore, polymicrobial infections may require multiple strategies to be simultaneously employed [71]. In any case, neutrophils cause inflammation of host tissues at the infection site, owing to their aggressive effector functions, such as the release of NETs, toxic granules, reactive oxygen species (discussed below) and proinflammatory cytokines into the extracellular space [40].

### 3.2. Neutrophils kill *Tv* using trogocytosis

We recently developed an *in vitro* co-culture system to assess neutrophil killing of *Tv,* using neutrophils purified from peripheral blood of healthy donors, and found that neutrophils of all 20+ donors tested rapidly and efficiently killed *Tv* [35]. We then investigated the mechanism(s) that neutrophils used to kill *Tv* in this system and took a process-of-elimination approach in which we inhibited each of the three mechanisms described above and assessed the ability of the neutrophils to kill *Tv* in their absence. To block the effects of NETosis, we used DNase; to determine whether extracellular degranulation was involved, we stimulated neutrophils and *Tv* in separate chambers of a trans-well plate, allowing the granule components of stimulated neutrophils to diffuse across the wells, but precluding cell–cell contact; to inhibit phagocytosis, we used the classic engulfment inhibitors cytochalasin D and wortmannin to impair actin polymerization and PI3 K signalling, respectively. We were surprised to find that while our data did not support a role for NETosis or extracellular degranulation in this rapid killing *in vitro*, our data pointed to phagocytosis as the mechanism, because killing was inhibited by the engulfment inhibitors [35]. However, the size of *Tv* would appear to preclude the use of phagocytosis, as *Tv* (10–15 µm diameter) [72] are larger than neutrophils (average 8.85 µm diameter) [72].

On closer examination using live imaging techniques, we found that neutrophils use a fourth, previously uncharacterized antimicrobial mechanism called trogocytosis (trogo = to nibble) to kill *Tv* [35]. In trogocytosis, neutrophils surround and ‘nibble' a target, often until the membrane is breached **[**73]. We used three-dimensional live confocal and four-dimensional super-resolution live confocal microscopy to observe neutrophils swarming individual trichomonads and ingesting small pieces of their membranes. In these experiments, we used propidium iodide, a membrane-impermeable nucleic acid sensor to track *Tv* viability in real-time during interaction with neutrophils and found that *Tv* were viable until multiple fragments of *Tv* membrane accumulated in the neutrophils. On average, parasites could survive 7 min after trogocytosis commenced and until 3–8 ‘bites' were taken, although we did observe variability. Furthermore, this was a process that neutrophils performed in aggregate, with an average of 3–6 neutrophils being present in the swarm during the killing, often nibbling from different angles [7,35]. We therefore hypothesize that *Tv* death occurs when a sufficient amount of membrane has been removed from the parasite's surface, or when the number/rate of ‘bites' overwhelms membrane repair machinery.

It was reassuring to find that our observations, made using modern imaging technology, corroborated observations from decades ago*.* Rein *et al.* [23] observed multiple neutrophils swarming one trichomonad and ingesting fragments of the parasite, although the term ‘trogocytosis' was not yet coined and technologies were limited to strengthen the author's confidence that this was indeed a novel process. The authors observed that ‘trichomonads usually escaped from single [neutrophils],' [23, p. 577] similar to our observations that multiple neutrophils attack a single trichomonad at different angles, presumably to trap the motile parasite from swimming away.

Matlung *et al*. [73] also recently reported neutrophils performing cytotoxic trogocytosis against cancer cells, further supporting that neutrophils have a trogocytic mechanism in their arsenal against targets. In this study, researchers found that trogocytic killing was independent of neutrophil degranulation, as neutrophils from human donors with a genetic degranulation deficiency were still competent to kill cancer cells [73]. We ruled out contact-independent extracellular degranulation as a mechanism used by neutrophils to kill *Tv* because of the inability of stimulated neutrophils to inflict damage to *Tv* across a trans-well membrane insert [35]. However, it is not yet known whether the granules being released directly at the junction between neutrophils and *Tv* during trogocytosis could be aiding in the degradation of the parasite membrane into ‘bites.' Interestingly, we found that serine proteases are required for trogocytosis and killing of *Tv* [35]. While serine proteases are present at the neutrophil plasma membrane, they are also a component of neutrophil toxic granules, pointing to a potential role for granule exocytosis in neutrophil trogocytic killing. Future experiments will determine if neutrophil toxic granules have a role in neutrophil trogocytic killing of *Tv*.

Additionally, Matlung *et al*. [73] used electron microscopy and intravital imaging to generate very high-resolution images of trogocytosis in action and demonstrating that neutrophil trogocytic killing of cancer cells occurs *in vivo*. Therefore, this study provides evidence that neutrophil trogocytic killing of targets occurs *in vivo*. While, we are currently not able to test whether trogocytosis of *Tv* occurs *in vivo* owing to severe limitations of *Tv* mouse models, the Matlung study increases our confidence that the observation of trogocytosis is not an *in vitro* artefact. Still, neutrophil killing of *Tv* during trichomoniasis infection *in vivo* may be more complicated than *in vitro* systems can capture. While NETs did not play a role in our *in vitro* model of rapid killing (10 min – 2 h), it is possible that they could be employed at a later stage in some challenging infections where parasites are in microcolonies or enmeshed in biofilms with other organisms [74]. Mouse models that can recapitulate trichomonas-associated pathology and neutrophil recruitment, especially in the context of a humanized CVM, would be powerful tools in illuminating a more complete picture of neutrophil killing of *Tv* during infection. Still, the efficiency with which trogocytic killing occurs *in vitro* points to an important role for this process in infection control.

The term trogocytosis was coined in 1990, to describe the ‘nibbling' of rat neuronal cells by the brain-eating amoeba *Naegleria fowleri,* which was distinct from phagocytosis (phago; devour), in that only small fragments of the target cells were ingested, rather than being eaten whole [75]. In 2003, the term was again used to describe membrane and protein exchange that occurs during antigen presentation at the immunological synapse from an antigen-presenting cell to a T-cell [76,77], although in this case the trogocytosis did not result in the death of the target cell. Cellular nibbling or gnawing of one cell on an adjacent cell has now been observed across animals and amoebozoas, and during many different biological scenarios such as development, neural remodelling, infection, and mounting and executing immune responses [78]. Importantly, trogocytosis does not always lead to the death of the trogocytosed cell, so it may be helpful to begin to classify trogocytic processes into cytotoxic trogocytosis and non-cytotoxic trogocytosis. Matlung *et al*. [73] have proposed the term ‘trogoptosis' to describe the death of a target cell following trogocytosis. The first demonstration of cytotoxic trogocytosis was the observation that the parasite *Entamoeba histolytica* kills host cells using trogocytosis [79]. Furthermore, while membrane transfer always occurs during trogocytosis, it is not clear if cytosol is also exchanged in each case, as methods to detect small amounts of cytosol uptake with confidence do not exist. Matlung *et al.* [73] observed a reduction in cytosolic signal in the target cell following trogocytosis, indicating cytosolic transfer, and Ralston *et al*. [79] observed cytosolic transfer in 90% of trogocytic instances using human cells and *E. histolytica*. However, while we did observe some ‘bites' of *Tv* membrane containing *Tv* cytosol in neutrophils following trogocytosis, not all ‘bites' were observed to contain it, and we were not able to confirm that our detection method was sensitive enough to say with confidence that not all bites contain cytosol (F. Mercer, P. J. Johnson 2018, unpublished observation). Therefore, it is currently unknown whether cytosol transfer generally occurs during cytotoxic trogocytosis.

Another interesting observation about trogocytosis is that it appears to occur only on live cell targets, while the same targets would otherwise be phagocytosed if they were already dead. Amoebic trogocytosis was demonstrated to occur exclusively on live cell targets, whereas amoeba phagocytosed pre-killed, intact cells [79]. We similarly found that dead-intact trichomonads were engulfed whole, via phagocytosis [35], and work from others showed that despite the parasite being slightly larger than neutrophils, phagocytosis is possible under conditions in which it is forced, such as using centrifugation of neutrophils and parasites together [23,80]. In the case of neutrophil trogocytosis of *Tv*, one hypothesis is that the parasite's motile nature contributes to the evasion of phagocytosis, necessitating trogocytosis. However, in the case of *E. histolytica* trogocytosis of human cells, the targets were not motile. Therefore, it appears that amoeba, and potentially also neutrophils, may have sensing mechanisms by which they can assess whether a target should be phagocytosed or trogocytosed. However, currently the mechanism by which neutrophils are activated to specifically undergo trogocytosis is not characterized. Serum opsonins appear to play a role in initiating the process, as we will discuss below, however, serum opsonins also play similar roles in phagocytosis, so it is not yet known what differentially regulates the two processes.

### 3.3. A potential role for reactive oxygen species

When neutrophils become activated by a pathogen, reactive oxygen species (ROS) are produced and released from the cell and into the phagosome, in a process known as oxidative burst [81]. ROS in the phagosome helps to damage ingested pathogens, and ROS in the extracellular space can damage extracellular pathogens, but can collaterally damage host cells as well [82,83]. Activation of the ROS pathway is also involved in NET release [84]. Therefore, neutrophil ROS participate in the other neutrophil killing mechanisms; however, it is not known whether they play a role in trogocytic killing of *Tv*. Matlung *et al*. [73] found that neutrophil trogocytic killing of cancer cells was not affected in the absence of ROS generation, as neutrophils from patients with genetic deficiencies in ROS production were still able to kill cancer cells via trogocytosis, and as killing proceeded in the presence of diphenyleneiodonium (DPI), a chemical inhibitor of ROS production, suggesting that neutrophil trogocytic killing does not require ROS. However, studies report contradicting results on the role of neutrophil ROS in the killing of *Tv*. Rein *et al.* [23] concluded that neutrophil ROS production played a role in killing trichomonads *in vitro*, because the killing of *Tv* by neutrophils was reduced in the presence of catalase or superoxide dismutase, enzymes that break down intermediates in the ROS pathway, and because ROS could be detected at the trichomonad-neutrophil interface using biochemical methods. However, in the same study, the researchers also found that neutrophils isolated from patients unable to synthesize ROS were able to kill *Tv*, confounding their results [23].

We observed no reduction in *Tv* killing by neutrophils in the presence of catalase [35], supporting that ROS does not play a role in trogocytic killing. These inconsistent results may be attributed to the strains of *Tv* used in the experiments, as *Tv* has been reported to produce anti-oxidants to guard against ROS [85]. Oxygen concentrations during the assays could also contribute to discrepancies, as we performed our assays in a standard 5% CO_2_ incubator, while Rein *et al*. [23] performed their catalase inhibition experiments in open air [35]. Hypoxia (low oxygen) has been found to be associated with lower levels of neutrophil activity [86]; however, anaerobic conditions may be more physiologically relevant, particularly during infection [87]. Vaginal oxygen levels are highly variable, further confounding decisions about what an appropriate level would be to conduct these experiments [88]. Therefore, the contribution of ROS to killing may also best be assessed in an animal model.

## 4. The role of opsonins in *Tv*-neutrophil interaction

While we observed that neutrophils kill *Tv* using trogocytosis, the subcellular and molecular players in the trogocytic process are under-characterized. However, one of the clearest results observed by numerous groups studying *Tv* interaction with neutrophils is that in the absence of human serum, killing was completely abolished [35,73,80], pointing to an important role for serum opsonins in neutrophil trogocytic killing of *Tv*. Opsonins are serum proteins that coat pathogens, allowing cross-linking of a cell bearing an opsonin receptor to the ‘opsonized’ pathogen [23,35]. Neutrophils express opsonin receptors [89–91], thus opsonins can enhance contact between neutrophils and pathogens and act as tags for phagocytosis and trogocytosis [73]. Opsonins include antibodies and complement proteins [62].

### 4.1. The role of antibodies in neutrophil killing of *Tv*

Antibodies are produced in response to specific pathogens, and bind those pathogens or their components with high specificity. Antibodies also contain an Fc domain that can bind to Fc receptors on neutrophils or other immune cells, thus mediating opsonization [24,62,91]. While long-term immunity against *Tv* seems tenuous [92,93], evidence indicates that antibodies against the parasite and its components are formed during infection with *Tv*. First, serum derived from trichomoniasis patients had bright reactivity against *Tv* [49]. Furthermore, immunoglobulin M (IgM) and immunoglobulin G (IgG) antibodies were detected in *Tv*-infected patients in the vagina and endocervix, indicating that *Tv* elicits antibody production at the infection site [94,95]. Animal models of intravaginal trichomonad infection also show IgM, IgG and IgA responses [96,97].

Many of the early researchers studying the interaction of neutrophils with *Tv* emphasized that antibodies were not necessary for trichomonacidal activity because the killing observed was unaffected by adsorption of serum to *Tv*, to deplete any antibodies that may have bound to the parasite [23,80]. However, these studies used serum from individual healthy donors with no history of STI's [23], or no history of sexual activity [80]. Therefore, antibodies were unlikely to contribute to the killing measured in these studies, and therefore no change would be expected when antibodies are blocked.

Our studies are in agreement that neutrophils can kill *Tv* in the absence of antibodies; however, our data support that killing is enhanced by up to twofold if specific antibodies are present. We found that opsonization mediated by antibodies had a role in neutrophil killing of *Tv*, as trogocytic activity and parasite killing was reduced by about half when Fc receptors were blocked [35]. In these experiments, we used human serum from a commercial source, which was pooled from hundreds of male donors, of which several had probably encountered *Tv*, owing to the high prevalence of *Tv* in the general population. Furthermore, we did indeed confirm that antibodies which could bind to *Tv* were present in our commercial serum batch, using flow cytometry [35]. A role for antibodies in neutrophil trogocytic targets was also confirmed for neutrophil killing of cancer cells. When Fc receptors were blocked, Matlung *et al.* [73] observed a reduced trogocytic activity against cancer cells. Therefore, antibodies probably play a role in neutrophil trogocytic killing of *Tv*; however, the specific Fc receptors involved, and the full contribution of antibody to killing *Tv in vivo* remains to be determined.

### 4.2. The role of complement in neutrophil killing of *Tv*

The complement system is a group of reactive proteins found constitutively in human serum that bind to pathogens and mediate various downstream effector functions in pathogen clearance [62]. One major function of complement is to opsonize pathogens with fragments of complement proteins, which can cross-link to complement receptors on neutrophils and other immune cells. The major complement protein involved in opsonization is called iC3b [62].

Complement proteins have long been known to bind *Tv*, as other downstream effects of complement, such as direct parasite lysis have been observed in some circumstances [23,49,98]. Furthermore, several groups have demonstrated an essential role for the complement system in neutrophil killing of *Tv* because heat-inactivation of serum (which inactivates complement proteins), reduced killing [23,80,98]. While several groups have shown that complement can be activated spontaneously on the parasite surface [49,80,98], using a pathway called the alternative pathway of complement activation, one group showed that the presence of anti-*Tv* antibodies can also enable complement to be activated through an antibody-enhanced process called the classical pathway of complement activation [80]. These data further support that while antibodies are not necessary for anti-trichomonal host responses, they can enhance them. We have shown that iC3b coats the surface of *Tv* that have been incubated with human serum, pointing to complement opsonization as a method by which neutrophils are activated to trogocytose the parasite [35].

To mediate opsonization, iC3b can bind to receptors CR1, CR3, CR4 and C1qR [90]. Matlung *et al*. [73] showed that CR3 is required for neutrophil trogocytic killing of cancer cells; however, the specific receptors involved in killing *Tv* are not yet defined. Discerning the roles of antibody and complement in opsonizing *Tv* for trogocytic killing are important, as complement is a function of the innate immune system, while antibody develops after a first encounter with the pathogen [62]. Therefore, identifying which players are required and which specific roles they play in neutrophil trogocytosis will help to define when *Tv* can be cleared upon initial infection and when stronger recall responses are necessary.

## 5. *Tv* evasion from neutrophils

Importantly, clinical observation shows that many patients do not clear *Tv* on their own, but require antibiotic therapy, pointing to an inability of neutrophils to effectively clear the parasite during infection, and implicating that *Tv* employs neutrophil evasion strategies. In the subsections below, we present evidence that several neutrophil evasion strategies may exist. However, as the data is collected from reductive *in vitro* models, the effectiveness of each strategy remains to be tested in conditions that more closely resemble natural infection.

### 5.1. ‘Running or hiding’

In live imaging studies that we performed on neutrophils and *Tv*, we always vortexed parasites prior to adding them to the imaging platform to break up clumps, which would otherwise make it very difficult to visualize trichomonads interacting with neutrophils in a steady plane-of-view [35]. In these experiments, we always saw several neutrophils swarm around individual parasites, often attacking from all sides, and on average, 3-6 neutrophils were present in a swarm around a parasite [7,35]. However, many strains of *Tv* grow in clumps, and during infection, this clumping behaviour may facilitate the formation of microcolonies [21]. In fact, some strains that demonstrate higher pathogenic behaviours *in vitro* tend to clump more [99,100], pointing to clumping as a virulence trait of the parasite. As clumps of *Tv* would make it very difficult for multiple neutrophils to surround individual trichomonads, it is probable that clumping behaviour facilitates evasion from trogocytosis. Furthermore, if parasites on the outside of the clump are trogocytosed and killed first, it may take neutrophils longer to reach the parasites on the inside, giving those trichomonads longer to employ some other potential evasion strategies described below.

In addition to protective clumping behaviour, *Tv* may also repel away from neutrophils during infection. One study shows evidence that *in vitro*, trichomonads avoided travelling towards neutrophils by repelling away from ROS products produced during neutrophil oxidative bursts. In a chemotaxis assay using a plate in which trichomonads were separated from neutrophils by a filter, fewer trichomonads migrated into the filter when neutrophils were stimulated compared to when they were not [27]. One alternative interpretation of these results could be that conditions in which neutrophils were activated resulted in lower parasite viability, and thus fewer parasites able to chemotax at all. However, our results using trans-well assays with neutrophils and *Tv* separated by a filter demonstrate that *Tv* cannot be killed by soluble factors from activated neutrophils [35]. Therefore, we support the interpretation that *Tv* were repelled from activated neutrophils in these assays. Chemorepulsion by *Tv* depended on the dose and the type of stimulus on the neutrophils. To determine which antimicrobial molecule the stimulated neutrophils were producing to induce chemorepulsion, the stimulated neutrophils were treated with catalase or superoxide dismutase to break down oxygen metabolites. The results demonstrated chemorepulsion by *Tv* was induced by neutrophil ROS products [27]. However, as these assays demonstrated chemorepulsion within 45 min, it is not clear whether chemorepulsion would aid in trichomonad escape of neutrophil trogocytosis, which is a rapid process often complete within 15 min. However, it is conceivable that in tissues with large numbers of activated neutrophils, trichomonads may use chemorepulsion to avoid areas where neutrophils have recently cast NETs that could possibly ensnare them. While neutrophil oxidative burst may enhance *Tv* repulsion, other studies demonstrated that it may also induce apoptosis in neutrophils.

### 5.2. Inducing neutrophil apoptosis

*Tv* has demonstrated the ability to induce apoptosis in neutrophils by activating ROS. While neutrophils are short-lived cells, apoptosis occurred significantly more in neutrophils incubated with *Tv* than neutrophils alone, when incubated for 12 h [101,102]. To confirm that the apoptotic pathway was induced by *Tv*, neutrophils, trichomonads and a caspase-3 inhibitor were cultured together [102]. Caspases are proteases that play a role in signalling programmed cell death; of the broad class of caspases, caspase-3 and caspase-8 facilitate apoptosis [103]. Addition of a caspase-3 inhibitor to the co-culture resulted in a reduction in apoptosis [102]. These results suggested that caspase-3 in neutrophils was induced by *Tv*, which led to premature apoptosis. Elevated levels of ROS can trigger apoptosis in neutrophils [104,105] and the addition of ROS inhibitor DPI reduced apoptosis in neutrophils triggered by *Tv* [101]; therefore, *Tv* induction of ROS from neutrophils is thought to be the mechanism of *Tv*-induced neutrophil apoptosis. However, as trogocytic killing of *Tv* is rapid (approx. 15 min), it is improbable that individual parasites are able to evade killing by inducing this neutrophil suicide, which takes up to 12 h to ensue. However, at a population level, this mechanism may impede neutrophil killing of parasites that take longer to approach because they are on the inside of a microcolony or within a polymicrobial biofilm [21]. However, as neutrophils are continually replaced from the blood during persistent infections, it is not clear what the impact of neutrophil apoptosis is *in vivo*.

### 5.3. Suppression of neutrophil recruitment

As described above, mediators from the parasite itself, as well as inflammatory products that *Tv* induces from epithelial cells, monocytes and other neutrophils, serve as chemo-attractants for neutrophils to home to the site of infection. The major neutrophil-recruitment factor is likely to be the cytokine IL-8, as it is released rapidly and abundantly from multiple cellular sources and facilitates both extravasation and homing to microenvironments within tissues. Interestingly, *Tv* was recently shown to produce and secrete exosomes, small extracellular vesicles that contain RNA and protein and can be uptaken by host cells [46,106]. In addition to increasing host cell susceptibility to parasite attachment, *Tv* exosomes also seem to have the ability to suppress neutrophil recruitment, as host epithelial cells that were pretreated with *Tv* exosomes showed a reduction in IL-8 response upon subsequent parasite encounter [46]. These data support a model in which parasites secrete exosomes at the infection site to prime more distal areas of the vagina for colonization, by promoting epithelial cell adherence, and decreasing the number of neutrophils homing to the area. Furthermore, in preliminary mouse models, the application of parasite exosomes to mouse vaginas 48 h prior to infection reduced gross inflammation and reduced the Th17 response, which is a type of immune response associated with neutrophils [107]. Therefore, *Tv* exosomes appear to have immunosuppressive properties *in vivo*.

### 5.4. Evading antibody-mediated trogocytosis

As described above, neutrophil trogocytic killing of *Tv* was shown to be partially mediated by antibodies [35]. However, *Tv* secretion products were shown to contain cysteine proteases that have the ability to degrade antibodies *in vitro* [108,109], pointing to a mechanism by which the parasite could degrade antibody and escape trogocytosis. Furthermore, we found *Tv* to be capable of killing lymphocytes, and to preferentially target B-cells [43], the producers of antibodies. The B-cell cytotoxic effects of *Tv* were mediated by both contact-dependent and soluble factors [43]. As B-cells can be detected in the cervicovaginal mucosa during infection [110,111], it is also conceivable that *Tv* could be impeding the formation of antibody responses in the local infection site.

### 5.5. Escape from neutrophil extracellular traps

In addition to behaviours that point to mechanisms of trogocytosis evasion, it is also possible that *Tv* could evade NETosis, a late-stage, final effort neutrophil killing strategy that could conceivably be a relevant killing mechanism in the context of *Tv* microcolonies or biofilms [21,112]. Interestingly, *Tv*'s symbiont *M. hominis* contains a virulence factor that may play a role in degrading NETs released by neutrophils [112]. An *in vitro* assay of activated neutrophils and *M. hominis* confirmed that NETs were released from neutrophils, but that they were degraded in the presence of *M. hominis*, and specifically owing to *M. hominis* gene MHOM_0730, which encodes for a surface lipoprotein with a nuclease domain [112]. Control studies demonstrated MHOM_0730's ability to degrade linear double stranded DNA (dsDNA), circular dsDNA and single stranded DNA [112]; therefore, one property of strains containing the *M. hominis* symbiont could be their increased resistance to neutrophil attack. However, as these strains are also more immuno-stimulatory [43,44] and likely to elicit increased neutrophil recruitment, the ultimate result of *M. hominis* presence on infection persistence is unclear. Nonetheless, as neutrophils are associated with inflammation regardless of whether they succeed in killing the parasite, we hypothesize that *M. hominis +* strains are more pathogenic. Furthermore, proteomic analysis of *Tv* secreted products revealed the presence of a DNase in the *Tv* genome [113]. While it is unclear whether this DNase is expressed during infection or whether it is competent to degrade NETs, this represents another exiting possible avenue by which *Tv* could evade neutrophil killing in some circumstances.

## 6. Conclusion and open questions

The interaction between neutrophils and *Tv* has been studied for over four decades since it was first noted that neutrophils are present in high numbers in vaginal discharge of trichomoniasis patients [23]. Expanding on the current knowledge of how the host handles *Tv* infection is important for the development of novel prevention and treatment options, especially in light of increased antibiotic-resistant *Tv* strains [1,114,115]. Neutrophils probably extravasate and home to the site of *Tv* infection following gradients of LTB4 secreted by both the parasite and the host, and IL-8 secreted by the host. Once at the infection site, neutrophils may kill the parasites; however, many of the effector functions of neutrophils can also cause inflammation in the host [35,40,98,101]. Both degranulation and NETosis release toxic granules into the extracellular space, potentially damaging the surrounding host tissue [69]. While phagocytosis and trogocytosis do not appear to involve the release of toxins into the environment, neutrophils still die en masse following the attack of pathogens using these mechanisms, and the resultant dead cell products could still elicit tissue inflammation. Furthermore, neutrophil activation to perform any of these mechanisms is also accompanied by the secretion of inflammatory cytokines such as IL-8, IL-6, IL-1*β* and TNF [26], which recruit other immune cells, and have broad inflammatory effects such as vasodilation. So far, the only killing mechanism that has been shown to be effective against *Tv* is trogocytosis, which requires cell–cell contact. Both complement proteins and antibodies seem to facilitate this contact, as blocking Fc receptors hinders trogocytic killing, and using complement-deficient serum failed to eliminate *Tv* [35,49]. *Tv* has also plausibly evolved to evade neutrophils in order to survive and strategies such as inhibiting neutrophil recruitment, repelling away from neutrophils, hiding in aggregates or biofilms, inducing neutrophil death, and thwarting various neutrophil effector functions may all contribute to *Tv* evasion of neutrophils. Certainly, neutrophil-*Tv* interaction is very dynamic. However, much remains to be determined about the subcellular and molecular particulars of neutrophil-*Tv* interactions, what the downstream immunological consequences are, how all of the discoveries made *in vitro* contribute to actual outcomes *in vivo*, and what contributions the vaginal microbiota and the parasite's own symbionts make to ultimate outcomes of the neutrophil-trichomonad struggle.

### 6.1. Molecular mechanisms of neutrophil trogocytosis of *Tv*

The recently discovered mechanism of neutrophil trogocytosis of *Tv* remains to be further studied. Thus far, it is known that trogocytosis is a contact-dependent process, though the determination of specific neutrophil receptors remains to be elucidated.

While antibodies and complement factors are implemented in cross-linking the neutrophil to *Tv* to initiate trogocytosis, whether any other adhesion factors are involved, and what specific molecules and organelles carry out the acquisition of *Tv* material by neutrophils is unclear. Our data showing that trogocytosis and parasite killing is reduced in the presence of a serine protease inhibitor indicates that neutrophil serine proteases play a role in the trogocytic process. However, which specific serine protease, which subcellular location it acts from, and which targets on the parasite that it attacks are not known. It is also possible that the serine protease acts on a host target, which is more directly involved in mediating the nibbling phenotype. Cysteine proteases are effectors of trogocytosis in *E. histolytica* [116], but the effect of proteases of neutrophil trogocytosis of cancer cells has not been tested and is instead thought to be purely mechanical, as a result of actin-myosin contraction [73]. As mentioned above, for several instances of trogocytosis, including neutrophil trogocytosis of *Tv*, the effector cell trogocytoses live cell targets, but phagocytoses the same cellular targets if they are dead, pointing to a signalling mechanism downstream of sensing live versus dead cells [35,79]. However, it is not known whether there are any trogocytosis-specific players function in neutrophil trogocytosis of *Tv*.

### 6.2. Downstream immunological consequences of trogocytosis

As neutrophils are usually the first immune cells that respond to an infection, their actions can have formative effects on how the subsequent immune response proceeds. Neutrophils can shape the tissue environment by cytokine secretion and tissue damage. However, how adaptive immune responses are formed following trogocytic killing of pathogens is unknown. While *Tv* material is detected in neutrophils following trogocytosis, the fate of these ‘bites' in unknown. In *E. histolytica*, trogocytosed bites of host cells fuse with lysosomes, and lysosomal degradation is required for sustained trogocytosis [117]. However, it is not known whether ‘trogosomes' containing *Tv* material become degraded by lysosomes or toxic granules, or whether the *Tv* material is subsequently loaded onto major histocompatibility complex class I or II, which would have implications for the formation of T-cell responses.

Furthermore, it is not known whether *Tv* material enters the cytosol of neutrophils, which would have implications for both antigen presentation as well as neutrophil cell death.

Macrophages undergo a process called pyroptosis, a highly inflammatory programmed cell death that is initiated by inflammasomes. Inflammasomes are complexes formed by cytosolic nod-like receptors (NLRs) and activate caspase enzymes [118]. Although it has been shown that macrophages are able to undergo pyroptosis when challenged with *Tv*, it is unknown if neutrophils do the same [119]. For this to occur, *Tv* material would have to enter the cytosol in order to activate the cytosolic NLRs. Alternatively, the material of either of *Tv*'s symbionts could potentially activate inflammasomes if it enters the cytosol. It is not known whether ‘trogosomes’ contain *Tv* material only, whether *M. hominis* and *Trichomonasvirus* are contained within ‘bites' as well, and whether any of this material can subsequently enter the cytosol.

### 6.3. Neutrophil heterogeneity

Recently, the primitive implication of neutrophils as dirty-bombs, sent to die at the site of infection, has been challenged. Intravital microscopy has recently revealed neutrophils responding to sterile injury and playing roles in tissue repair [120] and also travelling back out of inflamed tissues, in a process termed ‘reverse migration' [120,121]. Therefore, some neutrophils may have longer lasting and more restorative roles than once thought. Similar to the paradigm of the ‘classical' versus ‘alternatively activated' macrophage (clunkily termed M1 and M2), different subsets of neutrophils, N1 and N2, have also been proposed, and are nicely reviewed elsewhere [122]. N2 neutrophils and neutrophils that may actually have suppressive function, termed Granulocytic–Myeloid-derived suppressor cells (G-MDSC), are thought to play immunosuppressive roles in tumour microenvironments and may have more tolerogenic roles in regeneration and wound healing, and resolution of inflammation. These tolerogenic neutrophils may even have functions in antigen presentation [123]. However, it is not known whether N2 neutrophils play a role in responding to *Tv*, or whether both types of neutrophils can perform trogocytosis.

### 6.4. Contributions by *Tv*'s symbionts

Some important roles for symbionts *M. hominis* and *Trichomonasvirus* in stimulating immune cells have been recently revealed [61], but many previous studies that claimed to axenically prepare their *Tv* parasites might not have checked for these intracellular ‘hangers-on', particularly before 1986 in the case of *Trichomonasvirus* [60] and 1998 in the case of *M. hominis* [124]. Furthermore, while old literature often used strains collected from symptomatic patients, defined strains of *Tv* now exist as community resources on American Type Culture Collection (ATCC). It would therefore be potentially beneficial to the field if some experiments were repeated with more defined parameters, such as consistent strains, and comparing the conditions +/− either symbiont. With these variables under control, it may be possible to better understand neutrophil activation and response among different strains and from different microbial stimuli. In particular, it will be interesting to determine if there are differences in activating ROS, trogocytosis and NETs from neutrophils if strains harbour these symbionts that have been demonstrated to activate discrete PRRs. Researchers may then be able to make better predictions about outcomes during infection resulting from pathogenic strains of the parasite and whether the microbial symbionts contribute to pathogenesis *in vivo*.

### 6.5. Effect of the microbiome

Another exciting future direction will be to assess the effect of neutrophils during *Tv* infection depending on changes in the CVM. The human CVM is generally rich in lactobacillus species during the steady-state, but can become overgrown with pathobionts such as *Gardnerella vaginalis*, in a state known as BV [21]. The BV state is strongly correlated with *Tv* infection, although it is not yet known whether BV predisposes to *Tv* or vice versa. However, while lactobacilli decrease *Tv* colonization of epithelial monolayers [125,126], biofilms formed by the pathobionts increase adherence and killing of epithelial cells by the parasite [21], which presumably increases inflammation in the tissue. Therefore, we would predict increased neutrophil recruitment to tissues infected with *Tv* and in a state of BV dysbiosis. We also hypothesize that the BV biofilms may stymie neutrophil efforts to trogocytose parasites, thus the overall effect of this additional neutrophil recruitment may be more pathogenic than productive. However, these hypotheses remain to be tested. Importantly, mouse models that can re-capitulate good levels of parasite colonization, and ideally a humanized CVM would be powerful tools to test how neutrophil-*Tv*-microbiome dynamics ultimately play out during infection.

A wealth of *in vitro* data has characterized interactions between this highly prevalent human parasite and its most abundant host cell adversary, giving clues about how attacks are mounted in time and space, and the impacts of the parasite, the host and the microbial partners of each, on the disease. However, many open questions remain about the specific molecular players that participate, and the ultimate outcomes of these interactions. Delving further into these questions will aid in a better understanding of trichomoniasis that can inspire improvements in future prevention and treatment strategies.

## Data accessibility

This article has no additional data.
